# Complement Suppresses the Initial Type 1 Interferon Response to Ocular Herpes Simplex Virus Type 1 Infection in Mice

**DOI:** 10.3390/pathogens13010074

**Published:** 2024-01-13

**Authors:** Daniel J. J. Carr, Adrian Filiberti, Grzegorz B. Gmyrek

**Affiliations:** 1 Department of Ophthalmology, Microbiology, and Immunology, University of Oklahoma Health Sciences Center, Oklahoma City, OK 73104, USA; 2 Department of Ophthalmology, University of Oklahoma Health Sciences Center, Oklahoma City, OK 73104, USA; adrian.filiberti@obi.org (A.F.); gbgmyrek@gmail.com (G.B.G.)

**Keywords:** complement, herpes simplex virus type 1, cornea, cytokines, chemokines, interferon, leukocytes, ocular infection, cornea

## Abstract

The complement system (CS) contributes to the initial containment of viral and bacterial pathogens and clearance of dying cells in circulation. We previously reported mice deficient in complement component 3 (C3KO mice) were more sensitive than wild-type (WT) mice to ocular HSV-1 infection, as measured by a reduction in cumulative survival and elevated viral titers in the nervous system but not the cornea between days three and seven post infection (pi). The present study was undertaken to determine if complement deficiency impacted virus replication and associated changes in inflammation at earlier time points in the cornea. C3KO mice were found to possess significantly (*p* < 0.05) less infectious virus in the cornea at 24 h pi that corresponded with a decrease in HSV-1 lytic gene expression at 12 and 24 h pi compared to WT animals. Flow cytometry acquisition found no differences in the myeloid cell populations residing in the cornea including total macrophage and neutrophil populations at 24 h pi with minimal infiltrating cell populations detected at the 12 h pi time point. Analysis of cytokine and chemokine content in the cornea measured at 12 and 24 h pi revealed that only CCL3 (MIP-1α) was found to be different between WT and C3KO mice with >2-fold increased levels (*p* < 0.05, ANOVA and Tukey’s post hoc *t*-test) in the cornea of WT mice at 12 h pi. C3KO mouse resistance to HSV-1 infection at the early time points correlated with a significant increase in type I interferon (IFN) gene expression including IFN-α1 and IFN-β and downstream effector genes including tetherin and RNase L (*p* < 0.05, Mann–Whitney rank order test). These results suggest early activation of the CS interferes with the induction of the type I IFN response and leads to a transient increase in virus replication following corneal HSV-1 infection.

## 1. Introduction

The complement system (CS) is an ancient defense mechanism against microbial pathogens that has evolved over millions of years [1]. The central component common to the three different pathways within the CS is complement component 3 (C3), one of the earliest identified molecules of the CS [2]. C3 is a critical component of the innate and adaptive immune system contributing directly to host defense via enzymatic generation of C3b that leads to (i) the formation of C5 convertase and C5b to elicit the terminal pathway, the membrane attack complex [3], (ii) C3b tagging of pathogens leading to opsonization [4], and (iii) T cell activation through intracellular complement stores [5,6]. C3 also plays a role in the clearance of viruses by coating the virion facilitating proteasome degradation, and thus preventing replication [4]. Within the cornea, complement components C1–C7, properdin, and factor B were originally identified with corneal fibroblasts producing C3 and C5 [7,8]. All components of the CS have been identified, elicited by infection or injury ultimately leading to the activation of the lectin, alternative, and classical complement pathways [9,10,11]. However, the CS is under tight regulation through the expression of complement regulatory proteins that prevent complement activation under normal conditions in order to preserve the architecture of the ocular surface and maintain visual acuity [12,13]. 

Herpes simplex virus type 1 (HSV-1) is a common human pathogen that has an affinity for mucosal epithelium as a first line of replication prior to proximal spread to other cell types and distally to the peripheral and central nervous system. In the human host, over 1.8 million individuals present with HSV ocular disease annually, and it is the leading cause of infectious cornea blindness in developed countries [14]. In the case of the mouse cornea, HSV-1 replicates locally within the epithelial cells and upon access to sensory fibers that innervate the cornea, the virus will travel to the trigeminal ganglia (TG) via retrograde transport where it will locally replicate and spread via anterograde transport back to the cornea or adjacent sites or to the central nervous system [15]. The innate immune system comprised of myeloid-derived cells and soluble anti-viral factors are recruited and produced, respectively, in response to the initial HSV-1 insult. Specifically, neutrophils initially traffic to the cornea disrupting the highly organized collagen fibers that can result in significant pathology pending clearance of the virus [16,17]. Inflammatory monocytes/macrophages follow neutrophil infiltrate and contribute to the control of HSV-1 replication during the first 48–120 h post infection (PI) [18,19,20]. Tissue-resident hematopoietic-derived cells including macrophages, dendritic cells, and mast cells also contribute to resistance or pathology in the cornea [21,22]. In addition to myeloid cell infiltration, soluble factors produced by resident cell populations including type 1 and 3 interferons (IFN) elicit resistance to HSV-1 within the first 72 h PI [23,24,25]. Chemokines are also key molecules that play an indirect role in pathogenesis and recruitment of leukocytes early following cornea HSV-1 infection [26,27,28]. The outcome of the initial host response in the mouse cornea is a robust inflammatory response that can lead to scarring and denervation in severe cases [29,30,31,32].

HSV-1 is known to encode immune evasion proteins including glycoprotein C (gC) which binds to the C3 protein preventing complement-mediated lysis of virus-infected cells and blocking complement-mediated virus neutralization [33,34,35]. Antigen-specific IgG is an important defense against HSV-1, and complement contributes to its potency in terms of virus neutralization [36]. Previous research by our group found targeting complement through cobra venom factor, or specifically, using C3KO mice, preserved corneal nerve projections and mechanosensory function following HSV-1 infection, whereas in fully competent animals, denervation and sensation were lost in a time- and CD4^+^ T cell-dependent manner [37]. While infectious virus levels were not found to be different in the cornea measured at day three and day seven PI, complement assisted in the clearance of viral antigens and reduced the overall levels of inflammation, comparing WT to C3KO mice [37,38]. The present investigation was undertaken to extend these findings by assessing the immediate host response to cornea HSV-1 infection with a focus on CS and the type 1 IFN response as these two pathways represent ancient innate defenses [1,39]. 

## 2. Results

### 2.1. C3KO Mice Possess Less Infectious Virus and Express Lower Levels of HSV-1 Lytic Genes Compared to Wild-Type Mice 12–24 h Post Infection

**Figure 1:**
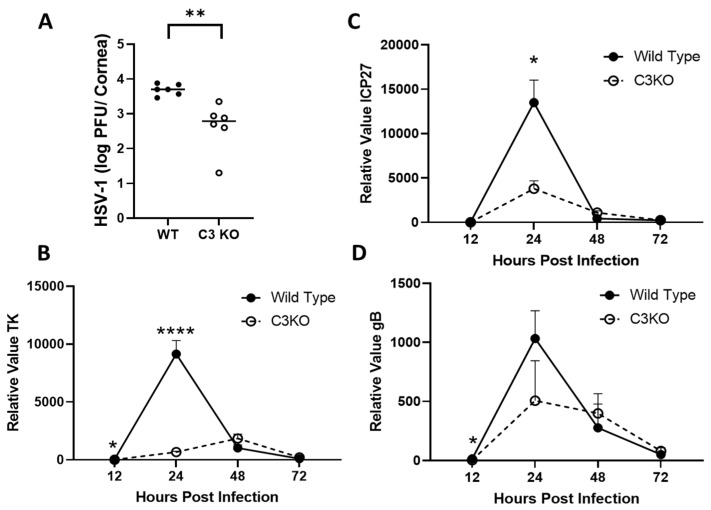
**HSV-1 replication is reduced in the cornea of C3KO mice**. (**A**) Titers of HSV-1 in the cornea of WT and C3KO mice at 24 h PI as determined by plaque assay. (**B**–**D**) HSV-1 lytic gene expression including (**B**) ICP27, (**C**) TK, and (**D**) gB at the indicated time point PI as determined by real-time RT-PCR. The mean ± SEM (n = 6–8 samples/time point) is plotted for each time point. Each graph is a summary of the results from 2–3 experiments. **** *p* < 0.0001, ** *p* < 0.01, * *p* < 0.05 comparing the two groups/time point as determined by Bonferroni–Dunn *t*-test method.

Complement components contribute to innate immunity including C6 that facilitates neutrophil phagocytosis and formation of extracellular traps [40]. We previously reported that the absence of C3 accelerated cornea pathology following HSV-1 infection, but this increase in corneal opacity and neovascularization was not due to changes in infectious virus recovered in the cornea 3–7 days post infection (PI) [38]. As the replicative cycle of HSV-1 is completed within 18–24 h PI, and our previous work did not investigate time points earlier than 72 h PI, a study was undertaken to determine if the absence of a fully functional CS altered the resistance landscape of the cornea to HSV-1. Therefore, wild-type (WT) and C3KO mice were infected with HSV-1 and assayed for infectious virus in the cornea 24 h PI. Notably, the results show that C3KO mice possessed significantly less infectious virus in the cornea compared to WT animals (Figure 1A). These results are consistent with lytic gene expression in that C3KO mouse corneas expressed less infected cell protein (ICP)27, thymidine kinase (TK), and glycoprotein (g)B at 12 and 24 h PI compared to WT mouse corneas (Figure 1B–D). However, at latter time points during acute infection (i.e., 48–72 h PI), similar levels of lytic genes were detected in the corneas of WT and C3KO, mice consistent with earlier reports of similar levels of infectious virus recovered in the cornea of WT and C3KO mice 72 h PI [38]. 

### 2.2. C3 Levels in the Cornea following HSV-1 Infection Peak at 12 h PI

**Figure 2:**
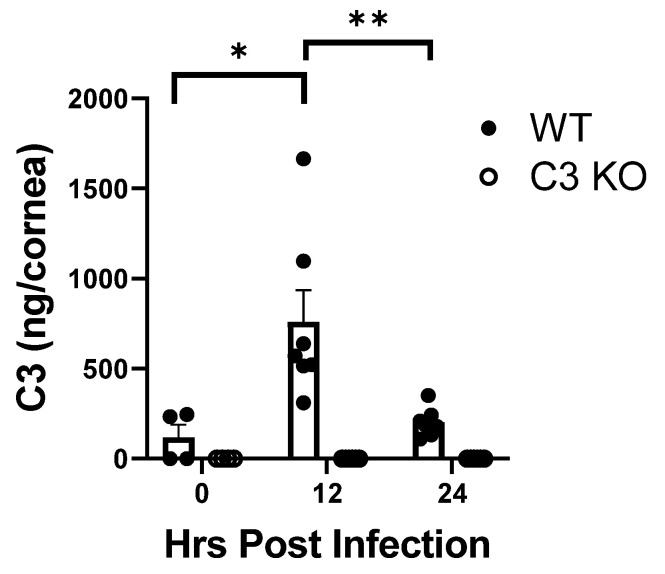
**C3 levels peak in the cornea of WT mice at 12 h PI.** WT and C3KO mice were infected with HSV-1 (500 PFU/cornea). At 12 or 24 h PI, the mice were exsanguinated and the corneas were removed and processed for C3 content by ELISA. The results are depicted as mean + SEM (n = 4–8 samples/time point) and plotted for each time point. The 0 h PI time point represents uninfected mice to serve as background control. The graph is a summary of the results from 2–3 experiments. ** *p* < 0.01, * *p* < 0.05 comparing the indicated groups as determined by the Holm–Sidak multiple *t*-test method.

C3 levels are normally detected in the cornea of uninfected mice and increase with a peak at D3PI during acute HSV-1 infection [37]. However, early time points have not been evaluated. Therefore, C3 levels were determined earlier at the 12 and 24 h PI time points. In comparison to uninfected controls, C3 levels increased dramatically 12 h PI but dropped down to near baseline levels by 24 h PI (Figure 2). As expected, there was no C3 detected in the C3KO mice (Figure 2). Thus, taking the previously published data [37] and those found herein, there appears to be two peaks of C3 with an initial peak at 12 h PI as a result of local resident production followed by a peak at 72 h likely due to the influx of leukocytes including neutrophils and monocyte/macrophages.

### 2.3. Myeloid Cell Infiltrate into the Cornea Is Similar between WT and C3KO Mice Following HSV-1 Infection

**Figure 3:**
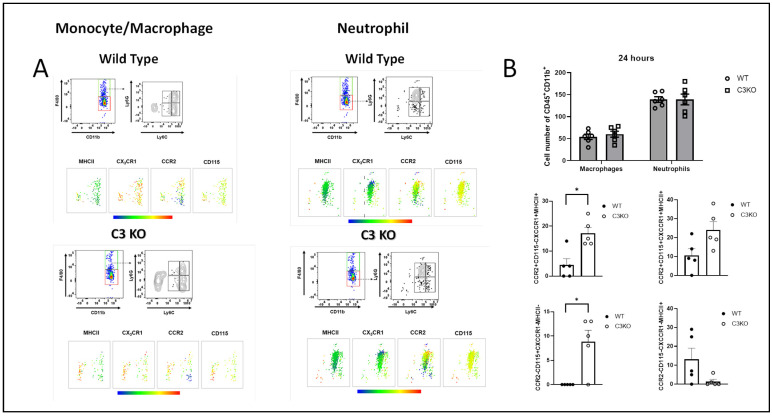
**Myeloid cell infiltration into the cornea of WT and C3KO mice does not differ at 24 h PI.** WT and C3KO mice were infected with HSV-1 (500 PFU/cornea). At 24 h PI, the mice were exsanguinated, and the cornea was removed and processed for myeloid cell content by flow cytometry. The gating strategy is shown for monocyte/macrophage and neutrophil populations in panel (**A**). Panel (**B**) shows the summary of myeloid cell types, n = 5–6/group, * *p* < 0.05 comparing the indicated group as determined by Mann–Whitney rank order test.

Previous results found inflammatory monocytes/macrophages are critical in the control of HSV-1 replication in the cornea [19]. We therefore investigated myeloid cell infiltration into the cornea over the first 24 h PI comparing HSV-1-infected WT to C3KO mice. Defining our inflammatory monocyte/macrophage population and the neutrophil population, (Figure 3A) we found near-equivalent numbers of cells residing in WT and C3KO mouse corneas at 24 h PI (Figure 3B). Likewise, the overall myeloid cell infiltration (CD45^+^CD11b^+^) was not significantly different between the two groups of infected mice at 24 h PI (Figure 3B). However, within the macrophage populations that were F4/80^+^Ly6C^−^Ly6G^−^, two subpopulations were significantly elevated in the cornea of C3KO mice at 24 h PI including those phenotypically defined as CCR2^+^CD115^−^CX_C_CR1^+^MHCII^+^ and CCR2^−^CD115^+^CX_C_CR1^−^MHCII^−^ (Figure 3B). Other macrophage subpopulations including CCR2^+^CD115^+^CX_C_CR1^+^MHCII^+^ and CCR2^−^CD115^−^CX_C_CR1^−^MHCII^+^ showed a trend for differences comparing the WT to C3KO groups, but they did not reach significance (Figure 3B). The 12 h PI time point revealed less than 100 total myeloid cells residing in the corneas of either WT or C3KO mice. While we have not accessed the activity of the cells in terms of nitric oxide or TNF-α production, whether there is any impact of the CD45^+^Ly6C^−^Ly6G^−^F4/80^+^CCR2^+^CD115^−^CX_C_CR1^+^MHCII^+^ population residing in the cornea and viral surveillance is not likely due to the modest number of these cells recovered in the cornea, suggesting other cells or pathways are involved in the control of cornea HSV-1 replication early PI.

### 2.4. CCL3/Macrophage Inflammatory Protein (MIP)-1α Is Elevated in the Cornea of WT Mice 12 h PI

**Figure 4:**
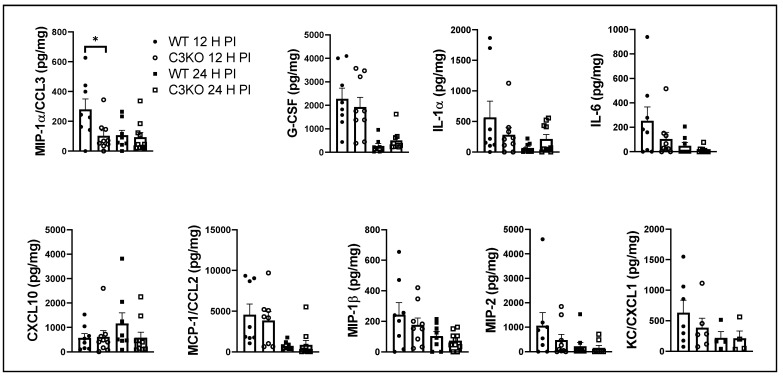
**MIP-1α/CCL3 is elevated early in the cornea of WT mice in response to HSV-1 infection.** WT and C3KO mice were infected with HSV-1 (500 PFU/cornea). At 12 or 24 h PI, the mice were exsanguinated, and the cornea was removed, homogenized, and clarified supernatant was assessed for analyte content by suspension array. Bars represent mean ± SEM, n = 8–10/group/time point from 3 experiments. * *p* < 0.05 comparing the indicated group as determined by ANOVA and Tukey’s post-hoc *t*-test.

There are a number of cytokines including TNF-α and IFN-γ that have been found to restrict HSV-1 replication and/or spread [41,42,43]. Likewise, chemokines are known to recruit cells from circulation (including monocytes and NK cells), and these cells contribute to resistance to HSV-1 infection. Therefore, corneas were collected from exsanguinated mice 12 and 24 h PI and assessed for cytokine/chemokine content by multiplex suspension array analysis. Of the 25 analytes assessed, only CCL3/MIP-1α was found to be significantly different between WT and C3KO infected mice and only at the 12 h PI time point (Figure 4). Other analytes detected including granulocyte-colony-stimulating factor (G-CSF), IL-1α, IL-6, IL-9, CCL2, CCL4, CXCL1, and CXCL10 were all similar between WT and C3KO mice at 12 and 24 h PI (Figure 4). Uninfected levels for these analytes in the WT (n = 5) and C3KO (n = 4) corneas were as follows: MIP-1α: 0.82 ± 0.82 pg/mg (WT) and 50.9 ± 24.0 pg/mg (C3KO); G-CSF: 6 ± 6 pg/mg (WT) to 372 ± 372 pg/mg (C3KO); IL-1α: 94 ± 94 pg/mg (WT) and 87 ± 60 pg/mg (C3KO); IL-6: 2 ± 2 pg/mg and 32.6 ± 20.3 pg/mg (C3K0); CXCL10: 6.5 ± 6.5 pg/mg (WT) and 109 ± 60.5 pg/mg (C3KO); MCP1/CCL2: 9.7 ± 9.7 pg/mg (WT) and 623 ± 623 pg/mg (C3KO); MIP-1β: 2.2 ± 2.2 pg/mg (WT) and 3.0 ± 3.0 pg/mg (C3KO); MIP-2: 10.4 ± 10.4 pg/mg (WT) and 112 ± 112 pg/mg (C3KO); and KC/CXCL1: 0 pg/mg for WT and C3KO mice. Thus, the levels of cytokine/chemokines detected in the cornea within the first 24 h PI did not correlate with either resistance to HSV-1 infection or recruitment of cells to the cornea.

### 2.5. IFN-β and Downstream Effector Molecule RNase L Are Elevated in the Cornea of C3KO Mice Early Post Infection

**Figure 5:**
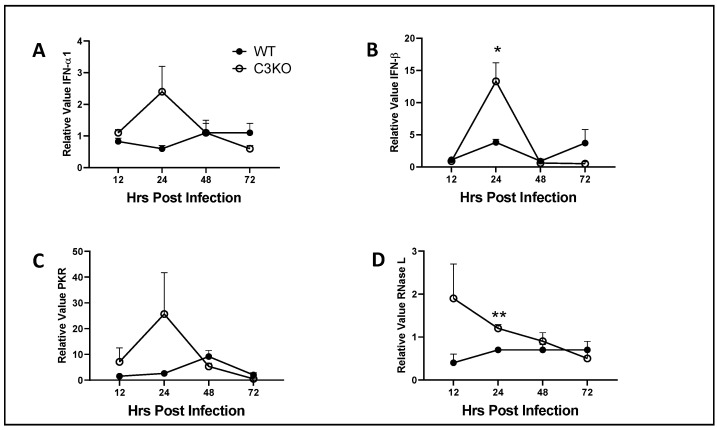
**IFN-β and RNase L expression are elevated in the cornea of C3KO mice early post HSV-1 infection.** C57BL/6 WT and C3KO male and female mice (n = 6–10/group/time point) were infected with HSV-1 McKrae (500–1000 PFU/cornea). At the indicated time PI, the mice were exsanguinated and the corneas were processed for mRNA analysis by real-time RT-PCR for relative expression of (**A**) IFN-α1, (**B**) IFN-β, (**C**) PKR, (**D**) RNase L. The results are expressed as the mean ± SEM relative value as determined using the ∆∆C_t_ method. ** *p* < 0.01, * *p* < 0.05 comparing the indicated groups at the indicated time point as determined by Holm–Sidak multiple *t*-test method.

Type 1 IFN including IFN-α and -β are potent, anti-viral cytokines that block replication of HSV-1 [23,44]. Likewise, some HSV-1-encoded proteins including ICP0, ICP27, ICP34.5, and VP16 [45,46,47,48,49] specifically target aspects of the type 1 IFN signaling pathway to block type 1 IFN activation or downstream effector molecule expression or activity. Therefore, we investigated the levels of expression of type 1 IFN in the cornea over the course of the 0–72 h PI. The results show an increase in IFN-α1 expression in the cornea of C3KO mice compared to WT controls at 24 h PI but the results did not reach significance (Figure 5A). No other time points showed any difference in expression. However, IFN-β expression was significantly increased in the cornea of C3KO mice compared to WT controls at 24 h PI with similar levels to WT mice after 24 h PI (Figure 5B). Since type 1 IFN genes were elevated in the C3KO cornea, we next investigated IFN-activated downstream effector molecule gene expression including double-stranded, RNA-dependent protein kinase (PKR) and endoribonuclease L (RNase L). PKR is a serine/threonine kinase that suppresses translation initiation by phosphorylation of eukaryotic initiation factor 2 and is targeted by HSV-1 ICP34.5 [50,51]. RNase L catalyzes the cleavage of single-stranded messenger and ribosomal RNA preventing protein production, resulting in apoptosis [52,53]. PKR expression was found to be increased in the C3KO mouse corneas at 12–24 h PI but did not reach significance compared to the WT animal corneas (Figure 5C). By comparison, RNase L expression was elevated at 12–24 h PI in the cornea of C3KO mice with noted significance at the 24 h PI time point compared to the WT controls (Figure 5D). Taken together, the data suggest the absence of a functional complement system allows for an early response of type 1 IFN and type 1 IFN-driven, anti-viral pathways to be expressed that correlate with early resistance to HSV-1 infection.

## 3. Discussion

In the present study, HSV-1 replication was found to be reduced early (12–24 h PI) in C3KO mouse corneas compared to WT controls following corneal infection as measured by viral lytic gene expression and infectious virus recovered in the tissue. These unanticipated findings suggested to us the possible interference between complement and complement-independent, anti-viral pathways that contribute to host defense early PI. As shown in Figure 2, C3 is upregulated very early PI and may contribute to complement opsonization of virions that might otherwise be detected by sensors that drive type I IFN production. One prominent sensor in the cornea that significantly contributes to resistance to HSV-1 is stimulator of IFN genes (STING) and the downstream effector molecule, tetherin [54]. STING is a target of HSV-1 through the induction of microRNA-24 which inhibits its translation [55]. In the present study, we investigated STING and tetherin expression and found they were upregulated in the cornea of C3KO mice at 12 h PI. However, the results did not reach significance (*p* < 0.07–0.08). The surveillance of additional anti-viral molecules including IFN-γ and TNF-α along with leukocyte subpopulations that infiltrate the cornea and contribute to resistance were not found to be different comparing WT to C3KO mouse cornea samples. The one caveat to this last statement concerns two subpopulations of macrophages defined as F4/80^+^Ly6C^−^Ly6G^−^CCR2^+^CD115^−^CXCCR1^+^MHCII^+^ and F4/80^+^Ly6C^−^Ly6G^−^CCR2-CD115+CXCCR1-MHCII- cells that were elevated in the cornea of C3KO mice. However, the impact of the observed cells in the range of 7–22 cells per cornea pair is questionable. The influence of NK cells, a cell type known to regulate HSV-1 infection in the cornea [21,56], has not been explored in this study. While prior research has established the significance of NK cells in controlling the infection, the specific infiltration dynamics of these cells during the early post-infection period, for example, within 12–24 h post-infection (PI), remain unreported. Moreover, the peak NK cell (NK1.1+CD3-) infiltration reportedly occurs on day 5 PI in C57BL/6 (WT) mice, with several hundred cells infiltrating the cornea [57]. Other pathways that may not involve leukocytes but could contribute to resistance include Toll-like receptor (TLR)7 activation previously shown to have enhanced activity in the production of IFN-α in the absence of C3 [56] and the potent set of type 1 IFNs.

The two most studied type 1 IFNs and HSV include IFN-α and IFN-β, although another type 1 IFN, IFN-ε, has been found to be instrumental against genital HSV-2 infection [57]. While there is only one IFN-β species, there are several mouse IFN-α subtypes that have varying degrees of anti-viral activity against HSV-1 replication [58]. We investigated the expression of IFN-α1 and IFN-β in the cornea of WT and C3KO mice from 12 to 72 h PI as a means to possibly correlate expression to lytic gene expression and infectious virus content. IFN-α1 and IFN-β were found to be elevated early in the C3KO mouse cornea in response to HSV-1, with IFN-β expression significantly elevated compared to WT mouse cornea samples. Whereas IFN-β shows greater anti-viral activity against HSV-1 compared to IFN-α1, both can antagonize HSV-1 replication through downstream effector molecules [59].

Two prominent downstream effector molecules induced by type 1 IFNs include RNase L and PKR [60]. Previous results reported PKR to be a principal molecule involved in resistance to HSV-1 replication in the trigeminal ganglia of mice transduced with IFN-β, whereas RNase L was responsible for resistance to HSV-1 infection in the cornea [61,62]. In the present study, PKR and RNase L expression were elevated in the cornea of C3KO mice with RNase L expression significantly different to levels found in WT cornea 24 h PI. Such results are consistent with the findings showing IFN-β expression is elevated in the cornea of HSV-1-infected C3KO mice at this time point as well. Other anti-viral pathways may also be impacted by the loss of C3 including interferon-induced transmembrane protein 1 (IFITM1), IFN-stimulated gene 15 (ISG15), and viperin, all of which possess potent action against HSV-1 infection [63,64,65]. Finally, the loss of C3 in the cornea and its impact on HSV-1 replication is limited since viral titers are similar to WT cornea samples by day three PI [38]. However, the loss of C3 may be more impactful within the peripheral and central nervous systems in which C3KO mice show an elevation in HSV-1 titers in the TG and brain stem that correlated with a decrease in cumulative survival [38]. Whether the CS influences anti-viral defenses in nervous tissue has not yet been determined but would be of considerable interest to pursue.

Cytokines and chemokines play an integral role in the innate immune response to ocular HSV-1 infection in the recruitment of leukocytes including neutrophils and monocytes/macrophages as well as dampening virus replication and spreading typically at the expense of irreversible tissue damage [27,66,67,68]. Previous experimental studies have found that the CS influences the outcome of ocular autoimmune diseases including the induction of specific cytokines/chemokines including IFN-γ and CXCL10 [69,70]. In the present study, the absence of C3 had very little impact on any of the 25 analytes measured, with the exception of MIP-1α/CCL3 which was found to be reduced in the cornea of C3KO mice at 12 h PI compared to WT animals. Early work reported that CCL3 contributed significantly to the development of herpes keratitis in HSV-1-infected mice, promoting the recruitment of neutrophils, whereas a later study suggested CCL3 did not significantly contribute to HSV-1-induced ocular pathology [28,71]. In the present study, the results suggest CCL3 did not contribute to the infiltration of neutrophils most likely due to the plethora of other factors expressed during these early time points that operate redundantly in the recruitment of leukocytes. Therefore, the results from the current study suggest the CS does not influence the overall content of cytokines/chemokines associated with ocular HSV-1 infection during the first 24 h PI. However, during the course of acute infection, an intact CS does dampen the inflammatory nature of ocular HSV-1 infection, reducing the expression of select chemokines, cytokines, matrix metalloproteins, and vascular endothelial growth factor A likely through facilitating the clearance of viral antigens [38]. As components of the CS include inhibitory factors that have pleiotropic functions outside the CS [13], more in-depth studies are required to establish the overall contribution of the CS in ocular HSV-1 infection, latency, and reactivation that would, undoubtedly, improve our understanding of microbial pathogenesis in the cornea.

## 4. Materials and Methods

### 4.1. Mice

C3KO mice (stock no. 003641 on a C57BL/6J background maintained as a colony at Dean McGee Eye Institute) and C57BL/6J (WT) male and female mice (6–8 weeks old) were obtained from The Jackson Laboratory (Bar Harbor, ME, USA). The mice were housed in a specific pathogen-free vivarium at the Dean A. McGee Eye Institute located adjacent to The University of Oklahoma Health Sciences Center. Mice were anesthetized for all procedures using an intraperitoneal injection of xylazine (6.6 mg/kg) and ketamine (100 mg/kg) and euthanized as described previously [29]. This study was conducted according to the approved protocol (21-095-CI) by the University of Oklahoma Health Sciences Center animal care and use committee.

### 4.2. Cells, Virus, and Viral Plaque Assay

Green African monkey kidney epithelial cells (Vero cells) were purchased from the American Type Culture Collection (Manassas, VA, USA) and used to grow HSV-1 McKrae strain at a stock titer of 1–3 × 10^8^ PFU/mL. Vero cells were propagated in Roswell Park Memorial Institute (RPMI) 1640 medium supplemented with L-glutamine, 10% fetal bovine serum (FBS), and antibiotics (complete media) (Thermo Fisher/Life Technologies Ltd., Grand Island, NY, USA). Corneas from mice infected with HSV-1 were removed at times PI and homogenized in 500 µL phosphate-buffered saline (PBS). The homogenized samples were centrifuged (10,000× *g*, 1 min), and the clarified supernatant was assayed for viral content by standard plaque assay [26].

### 4.3. Ocular Infection

C3KO and WT mice were infected with HSV-1 (500–1000 plaque-forming units [PFU]/cornea) following scarification of the cornea with a 25-gauge needle. Specifically, the needle was passed over the cornea surface 30 times horizontally and vertically. Any tear exudate was removed using a Kim wipe prior to applying the virus in a 3 µL volume of PBS. At the indicated time PI, the mice were exsanguinated by injecting 10 mL of PBS into the left ventricle of heavily sedated mice. Tissue was collected and processed as described below.

### 4.4. C3 ELISA and Cytokine/Chemokine Multiplex Suspension Array

Corneas from uninfected and infected mice were removed following exsanguination and placed in PBS containing 1X protease cocktail inhibitor (Calbiochem, EMD Millipore, Billerica, MA, USA) on ice. Corneas were then homogenized, and the homogenate was centrifuged at 10,000× *g*, 1 min at 4 °C. The clarified supernatant was collected and immediately frozen at −80 °C until it was analyzed for C3 content by ELISA (Abcam, Waltham, MA, USA) or a 25-plex cytokine/chemokine suspension assay (EMD Millipore, Burlington, MA, USA).

### 4.5. Real-Time Reverse Transcriptase (RT)-Polymerase Chain Reaction (PCR)

The cornea pairs of WT and C3KO mice were removed at the indicated time PI and placed in 2.0 mL tubes containing Trizol (Life Technologies, Carlsbad, CA, USA) to isolate RNA. cDNA was generated from the RNA template using an iSCRIPT cDNA synthesis kit (Bio-Rad, Hercules, CA, USA). Targeted genes were amplified using forward and reverse oligonucleotide primers by RT-PCR as indicated in Table 1:

Proprietary sets of forward and reverse oligonucleotide primers targeting GAPDH, IFNα1, IFNβ, PKR, and RNase L to amplify products were obtained from a commercial source (Bio-Rad). Gene expression relative values were determined using the ∆∆C_t_ method as described [58]. Amplification of targeted genes and analysis of data were carried out as previously described [72].

### 4.6. Flow Cytometry

The corneas of infected WT and C3 KO mice were collected from exsanguinated mice 24 h PI. Tissue was digested using Liberase TL (Roche, Mannheim, Germany) for 45 min at 37 °C. The treated samples were filtered through a 40 µm mesh cell strainer (Midsci, Valley Park, MO, USA) and washed with PBS containing 2% FBS (staining buffer; SB). Single-cell suspensions were stained with viability dye Zombie Aqua (Biolegend) for 15 min at room temperature. Next, after washing with SB, the cells were incubated in Fc blocking antibody (anti-CD16/32) followed by staining with an antibody cocktail containing Spark Blue 550-conjugated anti-CD45, phycoerythrin-conjugated anti-CD11b, Pacific Blue-conjugated F4/80, APC-Cy7-conjugated anti-Ly6C, Brilliant Violet 605-conjugated anti-MHC class II (I-A/I-E), Brilliant Violet 650-conjugated anti-CCR2, FITC-conjugated anti-CX3CR1, PE-Dazzle 594-conjugated anti-CD115, and PerCP-Cy5.5-conjugated anti-Ly6G (all from Biolegend, San Diego, CA, USA). Samples were incubated for 30 min on ice in the dark. Subsequently, the cells were washed twice with 2 mL of SB by centrifuging for 5 min at 300× *g*, then fixed with 1% paraformaldehyde. Finally, samples were acquired on an Aurora Spectral flow cytometer (Cytek Biosciences, Fremont, CA, USA). The settings for the acquisition were established according to the manufacturer’s guidelines. Spectral unmixing was performed using single-stained reference controls acquired under SpectroFlo software version 3.1 (Cytek Biosciences, Chicago, CA, USA), with each fluorochrome spectral pattern validated based on the reference provided by Cytek Biosciences. Samples were analyzed using FlowJo software, version 10.7.1 (BD Biosciences, Ashland, OR, USA).

### 4.7. Statistics

Statistical analysis of data was performed using Prism 8 software (version 8.0; GraphPad Software, La Jolla, CA, USA). Data were analyzed between groups using the indicated analysis for statistical significance, *p*-value < 0.05.

## 5. Conclusions

We identified C3 is significantly elevated within 12 h PI following HSV-1 cornea infection. The absence of C3 was found to augment type 1 IFN and downstream effector molecules (including RNase L and PKR) in the cornea in response to HSV-1 infection. This increase in expression correlated with significant resistance against early virus replication. There was no real difference in overall neutrophil or monocyte/macrophage influx into the cornea early (12–24 h) PI, suggesting that the control of virus replication in C3KO mice early PI may be the result of an elevation in the early activation of the type 1 IFN pathway.

## Figures and Tables

**Table 1:** Primer sequences for targeted virus and mouse genes.

Gene	Forward	Reverse
ICP27	5′-GCA TCC TTC GTG TTT GTC AT-3′	5′-ACC AAG GGT CGC GTA GTC-3′
TK	5′ATA CCG ACG ATC TGC GAC CT-3′	5′-TTA TTG CCG TCA TAG CGC GG-3′
gB	5′-TCT GCA CCA TGA CCA AGT G-3′	5′-TGG TGA AGG TCC TCC ATA TG-3′

## Data Availability

The data that support the findings of this study are available from the corresponding author upon reasonable request.
